# Towards Bi-Inclusive Policies: Suggestions Based on Research on Dutch Same-Sex Attracted Young People

**DOI:** 10.1007/s13178-016-0241-1

**Published:** 2016-07-18

**Authors:** Jantine van Lisdonk, Saskia Keuzenkamp

**Affiliations:** 1 Centre of Expertise on Sexual and Reproductive Health and Rights, Rutgers, Utrecht, Netherlands; 2 Department of Sociology, VU University Amsterdam, Amsterdam, Netherlands

**Keywords:** Bisexuality, Same-sex sexuality, Sexual orientation, LGBT policy, LGBT, Youth, Equality, Diversity, Sexual diversity, Biphobia, Mononormativity, Netherlands

## Abstract

Dutch national LGBT policies are not bi-inclusive and this study provides suggestions for improvement, based on empirical research. Attention for bisexuality in policy appears simply to pay lip service and to endorse the construction of sexual orientation as a hetero/homo binary. The outcomes of our survey (*n* = 1449) and in-depth interviews (*n* = 38) of Dutch same-sex attracted young people suggest that special attention for bisexual people is warranted. Compared to exclusively same-sex attracted participants, the equally both-sex attracted participants scored worse on openness about their sexual attraction, visibility discomfort, perceived acceptance, and suicide attempts. Unique issues for bisexual-identified young people were identified as follows: marginalization of bisexuality; difficulty expressing bisexuality, particularly in relationships; and a lack of bisexual or bi-inclusive communities. These issues were all related to the hetero/homo binary and mononormativity. Suggested implications for more bi-inclusive policies focus on awareness of marginalization and invisibility of bisexuality, biphobia, community and capacity building, and comprehensive sexuality and gender education. Furthermore, rather than policies focusing on sameness, a comprehensive diversity perspective on sexuality and gender offers more space for bisexuality. This may be particularly relevant for young people who are exploring their sexuality and developing a sense of their sexual self.

The Netherlands has a reputation for being one of Europe’s most tolerant countries as regards to homosexuality (Keuzenkamp and Kuyper 2013). However, heterosexuality remains the norm and victimization of lesbian, gay, bisexual, and transgender people (LGBT) is not uncommon (Keuzenkamp 2010; Keuzenkamp et al. 2012). Consequently, Dutch national policies continue to focus on increasing the legal equality and social acceptance of LGBT people in society (Maliepaard 2015a). A closer look at these policies reveals that non-heterosexuality is still predominantly conceptualized as homosexuality and is not bi-inclusive.

This study draws attention to the marginalization and invisibility of bisexuality in Dutch society, policy, and research. Marginalization of bisexuality occurs when people claim to be speaking about LGB people, while failing to address bisexuality or engage with specific issues that are relevant for bisexual people (Barker et al. 2012a). Bisexual people often remain invisible in society because people are assumed to be heterosexual or homosexual, based on the gender of their current partner (Barker et al. 2012a). These marginalization processes often operate unintentionally in a “taken-for-granted world” and socially exclude people who are not part of the (hetero)normative world (Kitzinger 2005, p. 478; Robinson 2012). Hence, marginalization is difficult to expose and challenge (Kitzinger 2005), yet is perpetuated when bisexual people and their problems remain silenced, unrepresented, and un-prioritized (Barker et al. 2012a).

Due to the marginalization and invisibility of bisexuality in Dutch society, specific issues bisexual people may face remain unnoticed or underreported. By presenting mixed methods empirical research findings on Dutch same-sex attracted (SSA) young people (ranging from exclusively same-sex attracted to equally both-sex attracted), we aim to determine which specific attention to bisexuality in policies is relevant and necessary. The empirical study focuses on a three-layered question: Whether there are differences in experiences between sexual attraction subgroups; whether SSA young people experience a lack of space for a bisexual identity and bisexual expressions; and which unique issues for bisexual-identified people can be identified. Based on these findings, we then put forward a number of suggestions for more bi-inclusive policies.

## Bisexuality in Research: Definition, Measuring, and Attention

Since the seminal work of Alfred Kinsey (Kinsey et al. 1948) and Fritz Klein (Klein et al. 1985), there is an academic consensus that sexual orientation consists of several dimensions, such as sexual attraction, self-identification, and sexual behavior, and that individual experiences may be different between dimensions and can change over time (Hegna and Rossow 2007; McDermott 2010; Saewyc et al. 2009; Savin-Wiliams 2005). Survey studies which have been inspired by these works commonly conceptualize sexual attraction as a continuum and provide several response options ranging from exclusively attracted to men to exclusively attracted to women. In these models, gender is commonly conceptualized as binary.

We recognize that gender is more complex and that gender identities are not limited to *man* and *woman*. Some people perceive gender as a continuum (e.g., mostly woman, partly man/partly woman), question or play with the concept of gender (e.g., genderqueer, ze). Theoretically, we agree with a group of leading bisexuality researchers in the UK, in which bisexuality is understood as “having attraction to more than one gender” (Barker et al. 2012a, p. 3). This means that people can see themselves as attracted to both men and women, as attracted mostly but not exclusively to one gender, as attracted to people regardless of gender, or as people who dispute the notion that there are only two genders to which they can be attracted (Barker et al. 2012a, p. 3).

While the theoretical conceptualization of sexual orientation as multidimensional and sexual attraction as a continuum are widely acknowledged among scholars, reporting on sexual orientation as a multidimensional concept is challenging in large-scale survey research among general populations. Researchers generally measure or report one dimension of sexual orientation. Gender is usually assessed as a binary. There are inspiring examples of assessing gender in bisexual, open-minded, and transgender communities, providing options such as “mostly/only female” (Barker et al. 2008), “partly male, partly female,” “not male and not female,” “I don’t know (yet),” or “other (specify)” (Doorduin 2015). Nevertheless, in general populations, assessing gender as non-binary is often still considered radical or raises questions among participants. At least, this is the case in the Netherlands.

In research, attention for bisexuality is still relatively scarce compared to homosexuality (Barker et al. 2012a, b; Oosterhuis and Lipperts 2013; Rust 2002). For example, researchers still amalgamate bisexual people with homosexual people since they do not recognize the relevance of distinguishing between these subgroups (Barker et al. 2012b; Oosterhuis and Lipperts 2013). On a positive note, more researchers have started to compare experiences of bisexual versus gay and lesbian people (Rust 2002).

This growing body of research has demonstrated that distinguishing between sexual orientation subgroups is relevant (Diamond 2008; Hegna and Rossow 2007; Oosterhuis and Lipperts 2013; Rust 2002; Savin-Williams 2005). Bisexual people appear to be in a more difficult situation or have more negative experiences in several ways. Compared to gay- and lesbian-identified people, bisexual-identified people are less open about their sexual orientation to people in their social network, report more internalized homonegativity, report more mental health problems, score higher on suicidality, and show lower LGB community identification and community involvement (Barker et al. 2012a; Cox et al. 2010, 2011; D’Augelli et al. 2005; Herek et al. 2010; Kertzner et al. 2009). While the picture for Dutch both-sex attracted and bisexual-identified young people is incomplete and inconsistent (Franssens 2010; Kuyper 2011, 2015a), Dutch studies of both-sex attracted adults show fairly similar outcomes (Kuyper 2011, 2013; Van Lisdonk and Kooiman 2012).

This literature demonstrates differences between sexual orientation subgroups, yet these studies generally do not take into account other factors which may explain these differences. It is therefore not clear whether differences remain after controlling for other relevant factors. For example, compared to their gay- and lesbian-identified counterparts, individuals who identify as bisexual are likely to be younger, are more often women, are less open to other people about their sexual orientation, consider their sexual orientation less important in their lives, and are less likely to have had a relationship with a same-sex partner (Cox et al. 2010, 2011; D’Augelli et al. 2005; Herek 2009; Kertzner et al. 2009; Rust 2002). Perhaps differences between bisexual and homosexual people on mental health or openness about their sexual orientation are strongly related to bisexual people being younger, being more often women, and not having had a same-sex partner. We aim to contribute to this body of knowledge and provide more robust comparisons by investigating whether any differences between sexual attraction subgroups remain present when other potentially relevant factors are taken into account.

A specific challenge which bisexual people may face is biphobia. This includes the denial of bisexuality as a sexual orientation, the invisibility and marginalization of bisexuality, and stereotypes such as being sexually promiscuous, a threat to families and relationships, and bisexual people taking the “easy option” (Barker et al. 2012a, b; Diamond 2008; Eliason 1997; McLean 2008; Rust 2002). Biphobia can be experienced from heterosexual as well as from gay and lesbian people (Barker et al. 2012a; Rust 2002).

While Herek observes that bisexual people report less discrimination compared to gay and lesbian people, he dismisses the interpretation that bisexuality is less stigmatized than homosexuality (Herek 2009). Rather, he concludes that the greater invisibility of bisexual people reduces the vulnerability to discrimination experiences (Herek 2009), which has also been reported in Dutch studies (Kuyper 2011; Van Lisdonk and Kooiman 2012). Several population-based studies have demonstrated that heterosexual people’s general attitudes towards bisexual people were less positive than towards gay and lesbian people (Eliason 1997; Steffens and Wagner 2004). So far, Dutch studies have only revealed small (Keuzenkamp and Kuyper 2013; Kuyper 2015b) or negligible differences in general attitudes (Van Lisdonk and Kooiman 2012). To our knowledge, biphobia among heterosexual, gay, and lesbian people has never been studied in depth in Dutch society. Nevertheless, what stands out is the invisibility of bisexual people in Dutch society, given that the prevalence of both-sex attraction and bisexual self-identification seems to be as common as an exclusively same-sex attraction or gay/lesbian self-identification in Dutch society (Keuzenkamp et al. 2012; Kuyper 2006, 2013).

## Lack of Representation of Bisexuality in Dutch National Policies

A brief review of the history of Dutch national policies shows that small steps have been taken towards addressing bisexuality, but that these have not been followed by a more bi-inclusive framing of same-sex sexuality. Since the first national policy plan in 1986, policies relating to the position of non-heterosexual citizens have focused on emancipation, legal equality, social acceptance, combating discrimination, and “anti-gay” violence (e.g., TK 1985/1986, 2007/2008, 2010/2011, 2012/2013). Until 2010, these policy plans targeted “homosexuals” and referred to “homo emancipation” or “lesbian and homo emancipation”.^1^ Since 2010, national emancipation policy documents have started to adopt the term “LGBT,” and it is presently politically correct in Dutch policy documents and formal language to refer to LGBT people and LGBT emancipation. However, explicit visibility of or attention for bisexuality is absent, thus reducing the use of the term to mere lip service, in which bisexuality is easily obscured under the umbrella of LGBT.^2^ For example, in recent years, “for the sake of readability” LGBT people were addressed as “homosexuals”, occasionally alternated with “homosexual and lesbian people” (TK 2010/2011). Main objectives were formulated as “equal rights for heterosexuals and homosexuals” (TK 2012/2013, p. 1, translation by authors) and “to improve the safety of LGBT people and to improve the social acceptance of homosexuality” (TK 2012/2013, appendix, p. 6).

Although current national policies do not devote specific attention to bisexuality, this is not to say that governmental attention for bisexuality is non-existent. The current minister has announced plans to explore whether national policies are sufficiently bi-inclusive. In addition, for several years, the government has provided financial support to organizations specifically targeting bisexual people. However, bisexuality remains largely invisible in policy plans and progress reports. There has been no fundamental shift towards bi-inclusive policies, and attention for bisexuality remains sporadic and superficial.

We agree with Maliepaard’s conclusion, based on an in-depth analysis of national emancipation policy documents, that bisexuality is not represented in these policies, because LGBT people are classed as a homogenous sexual minority group, with bisexual people being equated with homosexual people (Maliepaard 2015a). We argue that the absence of comprehensive attention for bisexuality is related to the underlying equality and rights-based approach of Dutch national LGBT policies. Robinson (2012) noted that in Dutch society, a sexual minority approach is strategically focused on sameness and normalization in striving for legal rights and social tolerance. Richardson and Monro (2012) described a similar approach in other Western countries. In such an approach, LGBT people need to be represented as a group of people who are “ordinary” and who are similar to each other. Sexual minorities are presented as a discrete group in the same way as ethnic minority groups, and sexual orientation is considered to be fixed, stable, and immutable (Herek 2000; Richardson and Monro 2012). A fixed sexual orientation, homogenization, and ordinariness of LGBT people are projected in these policies, but this may not necessary reflect how bisexual people consider themselves or how lesbian and gay people see bisexual people. In essence, such an approach often perpetuates the societal conceptualizations of sexual orientation as a hetero/homo binary, which makes invisible possible specific needs or problems of bisexual people and the need for policy that explicitly addresses bisexual specific issues.

## Present Study

This study focuses on the experiences of SSA young people as a basis for suggestions for more bi-inclusive policies. Sexuality is an important theme in young people’s lives in their transition to adulthood, and they may be particularly aware of social messages concerning sexuality and sexual orientation. Based on mixed methods data on Dutch SSA young people, we aimed to (a) investigate whether there are any differences in experiences between exclusively same-sex attracted, mostly same-sex attracted, and equally both-sex attracted subgroups; (b) assess whether they experience a lack of space for a bisexual identity and bisexual expressions; and (c) identify unique issues for bisexual-identified people. Informed by this empirical data, we discuss the outcomes of the three central questions in dialogue with each other and offer suggestions for more bi-inclusive policies.

The rationale for a mixed methods design was the ability to combine results based on quantitative and qualitative data strands to provide a more comprehensive picture. Each method was used to answer specific questions which led to complementary knowledge and a better overall insight (Creswell and Plano Clark 2011). Using quantitative data, we investigated whether there were any differences between sexual attraction subgroups in terms of openness about their sexual attraction, perceived acceptance, and perceived experiences of victimization, suicidality, visibility discomfort, and self-esteem. This was followed by additional multivariate analyses to determine whether differences remained significant after controlling for potentially relevant factors (age, gender, importance of their sexual attraction in their lives, degree of openness about their sexual attraction, and having relationship experience with a same-sex partner).

In this quantitative sample, we used sexual attraction as an indicator for participants’ sexual orientation. Here, we follow other scholars who have suggested employing this indicator in survey studies with a broad scope (Kuyper 2015a; McDermott 2010). Studies have shown that other sexual orientation dimensions may unintentionally exclude young people, since self-identification may be a very delicate issue for young people and young SSA individuals may not yet have had any same-sex sexual or relationship experiences (Kuyper 2015a; McDermott 2010). In this quantitative data analysis, we distinguished between equally both-sex attracted, mostly same-sex attracted, and exclusively same-sex attracted subgroups. The motivation to distinguish between three sexual attraction subgroups underscores our view that sexual attraction should be conceptualized as a continuum.

We used qualitative data to explore the views and experiences of young bisexual-identified and non-bisexual-identified SSA people on sexual orientation in general and bisexuality in particular. The combination of interviewing both bisexual-identified and non-bisexual-identified SSA people was conducive to revealing views and experiences concerning sexual orientation and related biphobia and marginalization of bisexuality. We also assessed whether the space for a bisexual identity was limited and whether bisexual-identified people felt they could openly express their bisexuality. Which bisexual expressions were tolerated and which were rejected? We also identified whether bisexual-identified young people faced unique problems which were not relevant, or less relevant, for the other SSA young people.

## Method

### Design, Sampling, and Data Collection

#### Mixed Methods Design

We used a sequential mixed methods design (Creswell and Plano Clark 2011). A quantitative Web-based survey was followed by in-depth interviews and samples were nested for the quantitative and qualitative data components. The quantitative and qualitative data were given equal emphasis in this study.

#### Sample and Recruitment

The study focused on SSA people aged between 16 and 26 years living in the Netherlands. The quantitative data collection was based on convenience sampling. To minimize the expected impact of a convenience sample on representativeness and selective non-response, the aim was to reach as many people as possible to fill in the survey. Multiple recruitment techniques were therefore used and special attention was paid to recruiting groups that are known to be difficult to recruit through traditional LGB channels, such as migrant and religious SSA people and non-LGB-identifying young people. It was important to include non-LGB channels, since research has shown that bisexual people do not always identify with or participate in LGB communities (Barker et al. 2012a). Participants were recruited through websites, newsletters, mailing lists, and social media targeting LGB people, particularly young LGB people, as well as young people in general. We included a few social media channels focusing on bisexual young women. To our knowledge, in 2009, there were no similar channels targeting bisexual young men. In addition, a promotional team recruited attendees at LGB-focused parties and events in a number of geographical regions in the Netherlands. All recruitment materials targeted same-sex attracted young people and labels such as homosexual, lesbian, gay, and bisexual were avoided.

Quantitative data collection was carried out using a self-administered online survey, which was placed online from May to July 2009 on a special website. A broad range of topics were addressed, including same-sex sexuality experiences, degree of openness about one’s sexual attraction, visibility discomfort, perceived acceptance and victimization, psychosocial well-being, and suicidality. After the quantitative data collection, participants were selected for in-depth interviews. We sought to achieve maximum variation in the qualitative sample in terms of gender, age, place of residence (rural or urban; geographically spread throughout the country), and sexual self-identification. An additional inclusion criterion was that participants were open about their sexual attraction to at least one person. Qualitative data collection took place between October 2010 and March 2011.

### Instruments for Quantitative Data Collection

#### Attraction

Participants were asked to indicate to whom they felt attracted on a 5-point scale (0 = Exclusively same sex, 1 = Mostly same sex, 2 = Attracted to men and women equally, 3 = Mostly other sex, 4 = Exclusively other sex). Two further options were also included (5 = I don’t know, 6 = Neither). We distinguished between three subgroups: exclusively same-sex attracted, mostly same-sex attracted, and equally both-sex attracted. Previous data analyses have shown that the mostly same-sex attracted group did not consistently resemble one of the other subgroups (Van Lisdonk and Van Bergen 2010) and we chose to provide more detailed information rather than clustering subgroups together. The participants who answered 3, 4, 5, or 6 were excluded from the analyses. The “Mostly other sex attracted” participants were excluded since studies focused on this subgroup show that this is a distinct subgroup (e.g., Savin-Williams and Vrangalova 2013), which prompted us not to combine them with the equally both-sex attracted group, while the subgroup was too small to report on separately.

#### Importance of Sexual Attraction

To assess whether participants’ sexual attraction was important in their lives, we posed the statement: “The fact that I feel attracted to men/women is important for who I am.” They were asked to rate their answer on a 5-point scale (0 = Completely agree, 4 = Completely disagree).

#### Degree of Openness

The degree of participants’ openness about their sexual attraction to others was measured using items that were mainly adopted from previous Dutch studies on young LGB people (De Graaf et al. 2005; Franssens 2010). Participants were asked whether people in their social network knew about their sexual attraction, measured separately with regard to their mother, father, extended family members, straight friends, and fellow pupils. “Don’t know” responses were coded as system-missing values. For the multivariate analyses, an index for the overall degree of openness was calculated for inclusion as a control variable, using the mean score for the categories that were applicable to them (e.g., not all participants had a mother). The index for the overall degree of openness ranged from 0 (Not out at all) to 2 (Out to all people). For this sample, Cronbach’s alpha = 0.79.

#### Same-Sex Partner

Participants were asked whether they had experience of having a relationship with a same-sex partner lasting at least 1 month.

#### Perceived Acceptance

Participants were asked whether their mother, father, extended family, straights friends, and fellow students had accepted that they felt same-sex attracted or both same-sex and other-sex attracted. For mother and father, responses ranged from 0 (Not accepted) to 4 (Completely accepted). In the other contexts, the response options were 0 (Yes), 1 (By some), 2 (No), and 3 (Don’t know). To compare perceived complete acceptance to lower levels of perceived acceptance, scores were recoded to 0 (Perceived limited acceptance) and 1 (Perceived complete acceptance). “Don’t know” responses were coded as system-missing values. In the analyses, participants were only included if they were open to some or all people in the specific context.

#### Perceived Experiences of Victimization

Measurement of participants’ experiences of victimization due to their sexual attraction was based on self-report; hence, we refer to perceived experiences of victimization. The degree of perceived experiences of victimization in the preceding 12 months was measured for the contexts of parents, extended family, straight friends, fellow students, and strangers (formulated as “People in the neighborhood/ unknown people”) with 1-item questions, for example: “Have you been victimized at your school during the past 12 months due to your same-sex attraction?” For each context, frequency scores were dichotomized into 0 (No) and 1 (Yes).

#### Suicidality

Lifetime suicidal ideation and suicide attempts were each measured with a single item on having suicidal thoughts and having attempted to end one’s own life. Frequency scores were dichotomized into 0 (No) and 1 (Yes).

#### Visibility Discomfort

Visibility discomfort refers to whether people are comfortably open about their sexual attraction to other people and in the public space. The degree of visibility discomfort was measured using an adapted version of the Public Identification as a Lesbian subscale. The original subscale consisted of 16 items and was part of the Lesbian Internalized Homophobia Scale (Szymanski and Chung 2001). In the adapted version, eight items were selected (items 2, 15, 19, 24, 30, 34, 41, 47) which we considered to be relevant for young people and which were appropriate for participants who did not identify themselves as gay or lesbian. Examples of this adapted subscale are: “When talking about my relationship to a straight person, I often use neutral pronouns so the sex of the person I am in a relationship with is vague,” and “If my peers knew that I feel attracted to men/women, I fear that a lot of them would not want to be friends with me.” For items that referred to having a same-sex partner, participants were asked to imagine having a same-sex partner. A 5-point Likert-type response scale was used, ranging from “Strongly agree” to “Strongly disagree.” For the multivariate analysis, the overall mean score was calculated. Higher scores indicated more discomfort with being open about their sexual attraction to others and in the public space. For this sample, Cronbach’s alpha = 0.87.

#### Self-Esteem

This was assessed using the Rosenberg Self-Esteem Scale (1979). The ten original items were recoded where necessary, and the overall mean score was calculated. Higher scores indicated higher self-esteem. For this sample, Cronbach’s alpha = 0.92.

### Instruments for Qualitative Data Collection

The first author conducted all the in-depth interviews at the locations preferred by the participants, on the condition that interference by other people would be kept to a minimum owing to the potentially sensitive topics to be addressed in the interview. The interviews lasted for 135 min on average; none was not shorter than 50 min. The interviews were semi-structured. The topic list included topics such as past and current sexual orientation experiences (sexual attraction, sexual behavior, relationships, self-identification); coming out; experiences of stigmatization due to their sexual attraction; and relations with heterosexual and LGB peers. Most questions had an open character, so as to invite participants to provide rich information and to leave space for them to mention things that were important to them. In addition, participants were presented with several statements relating to same-sex sexuality in order to prompt them to speak about their views on same-sex sexuality. One of the statements was: “Bisexuality is a stable sexual orientation.”

### Procedures

The study was approved by the internal review committee of the Netherlands Institute for Social Research and was registered with the Dutch Data Protection Authority. Prospective participants were given information about the study and its aims and content on the survey website as well as by e-mail prior to participating in the in-depth interviews. Participants were only eligible for inclusion in the qualitative sample if they had confirmed in the survey that they were willing to participate in follow-up studies and to be contacted by the researchers for that purpose. If necessary, the information about the study and the consent form were explained and discussed at the beginning of the interview. All participants provided written informed consent. Participants’ anonymity was guaranteed and participants were given pseudonyms.

### Data Analysis

Quantitative and qualitative data strands were analyzed separately, combining the findings to provide a comprehensive picture. In the quantitative data analyses, we investigated whether differences between exclusively same-sex attracted, mostly same-sex attracted, and equally both-sex attracted participants were present for a range of experiences: degree of openness, perceived acceptance, perceived experiences of victimization, suicidality, visibility discomfort, and self-esteem. Bearing in mind that differences between sexual attraction subgroups in sociodemographics and the visibility of their sexual orientation may affect outcomes, we conducted multivariate analyses to determine whether any differences in outcome variables remained present after controlling for other variables. Prior to the analyses of the outcome variables, we therefore tested differences between sexual attraction subgroups for potentially relevant variables which we had identified in previous studies: age, gender, importance of their sexual attraction in their lives, degree of openness about their sexual attraction, and having relational experience with a same-sex partner (see literature study in the previous section). Chi-square tests were used for categorical variables and one-way ANOVA for continuous variables. When tests were significant, variables were included as control variables in the multivariate analyses. Depending on the level of measurement of the outcome variables, we conducted logistic regression analyses or linear regression. To distinguish between sexual attraction subgroups, we used dummies for the mostly same-sex attracted subgroup and the equally both-sex attracted subgroup, with exclusively same-sex attracted subgroup as the reference group. Quantitative data analyses were carried out using SPSS version 17.0.

In the qualitative data analysis, major themes related to bisexuality and unique themes for bisexual-identified young people were identified, independently from the quantitative data analyses. All interviews were transcribed verbatim. Meta-themes for further analysis were identified based on the major topics that emerged from an initial analysis of the first 20 transcripts, as well as the logbook of the qualitative data collection phase and the researchers’ theoretical interests. These meta-themes were used to develop a first version of a codebook. Subsequently, all transcripts were coded and analyzed in Atlas.ti (version 6.2), using the a priori codebook which was developed further using inductive coding. The writing of memos during the coding process was conducive to generating and promoting transparency in the interpretation of findings (Friese 2012). This process of fine-coding was followed by axial coding in which codes were combined to generate subthemes. Participants were categorized as bisexual-identified or non-bisexual-identified based on their self-identification. The label “bisexual” is often abbreviated in Dutch to “bi.” In this article, to aid legibility, we consistently refer to bisexual, except in participants’ quotes.

### Participants

The quantitative study sample comprised 1449 Dutch SSA young people aged between 16 and 25 years. The mean age was 20.12 (SD = 2.79). The sample consisted of 839 exclusively same-sex attracted, 474 mostly same-sex attracted, and 136 equally both-sex attracted participants. There were more women (65 %) than men (35 %). Of the women, 12 % were equally both-sex attracted, 37 % mostly same-sex attracted, and 51 % exclusively same-sex attracted. Of the men, 4 % were equally both-sex attracted, 25 % mostly same-sex attracted, and 71 % exclusively same-sex attracted. Seventy-six percent of participants were enrolled in education and 71 % reported having a job. A minority of participants identified themselves as religious (22 %), predominantly Christian, which is comparable to the general population of Dutch young people. Sixteen percent of participants had a migrant background, meaning that at least one of their parents was born outside the Netherlands (Statistics Netherlands 2012).

The qualitative sample consisted of 19 women and 19 men aged between 18 and 26 years. Four women and three men identified as bisexual and felt attracted to women and men. In addition to these seven participants, two women and two men reported that they had experienced “a bisexual phase” in their lives. These four did not identify as bisexual. The non-bisexual-identified participants identified as homo(sexual) or lesbian, and about half of them primarily or also described their sexual orientation by saying “I like men/women”. Some described their sexual orientation as “I have/do not have a girlfriend/boyfriend.” Identifying as “gay” was rare and none identified as “queer,” “straight,” “questioning,” or “pansexual.”

In this sample, 21 participants were at school, 19 in work, 2 were unemployed, and 1 was chronically ill. Six participants had a migrant background, with one or both parents being of non-Dutch descent. Four of the participants considered themselves to be religious.

## Results

### Quantitative Differences between Sexual Attraction Subgroups

First, we determined whether potentially relevant variables were useful to include as control variables by testing for differences between sexual attraction subgroups. For the quantitative sample, Table 1 presents descriptive information of the possible control variables (age, gender, importance of sexual attraction, degree of openness, and having relationship experience with a same-sex partner) for each sexual attraction subgroup. Since sexual attraction subgroups differed significantly on each of these variables, these were all included as control variables in further analyses.

**Table 1:** Gender, age, and same-sex sexuality experiences compared between sexual attraction subgroups

	Exclusively same-sex attracted (*n* = 839)	Mostly same-sex attracted (*n* = 474)	Equally both-sex attracted (*n* = 136)	*F* or *χ* ^2b^	*df*	*p* value
Age (M (SD); range 16–25)	20.33 (2.77)	19.87 (2.79)	19.74 (2.78)	5.53	(2, 1446)	0.004
Gender (*n*; % women)	57.4	73.4	86.8	64.36	2	<0.001
Importance of sexual attraction (M (SD); range 0–4)	1.00 (0.96)	1.13 (0.98)	1.28 (0.92)	7.36	(2, 1446)	0.001
Openness^a^ (M (SD); range 0–2, low to high)	1.60 (0.51)	1.25 (0.65)	0.94 (0.70)	91.34^c^	(2, 339)	<0.001
Same-sex partner (*n*; % yes)	66.2	53.8	43.4	36.46	2	<0.001

^a^Due to a technical problem, there were some missing scores on the item about openness to their mother and father. The non-response group did not differ from the response group in terms of sociodemographics or same-sex sexuality experiences

^b^For the variables age, importance of sexual attraction, and openness, differences between sexual attraction subgroups were assessed using one-way ANOVA. For the variables gender and same-sex partner, *χ* ^2^ tests were used to assess differences

^c^Since Levene’s test indicated that variances were unequal, the Welch *F* ratio is reported

Table 2 shows the results for the outcome variables degree of openness, perceived acceptance, perceived experiences of victimization, and suicidality. Table 3 presents the results for visibility discomfort and self-esteem. In both tables, the first columns present descriptive results for each sexual attraction subgroup. Table 2 presents the odds ratios for the mostly same-sex attracted and equally both-sex attracted subgroups, based on logistic analyses. Similarly, Table 3 shows the unstandardized beta coefficients, based on linear regression analyses. In all logistic and linear regression analyses, we controlled for age, gender, importance of sexual attraction, degree of openness, and having relationship experience with a same-sex partner. Since degree of openness is highly correlated with the outcome variables openness, perceived acceptance, and visibility discomfort, we excluded degree of openness as a control variable in these specific multivariate analyses.

**Table 2:** Results for openness, acceptance, victimization, and suicidality

	Percentages^b^			Logistic regression analyses^c^	
Outcome variables	Exclusively same-sex attracted	Mostly same-sex attracted	Equally both-sex attracted	Odds ratio on mostly same-sex attracted vs exclusively same-sex attracted	Odds ratio on equally both-sex attracted versus exclusively same-sex attracted
Open to					
Mother (yes %, *n* = 1083)	91.8	76.6	55.6	0.33***	0.11***
Father (yes %, *n* = 1056)	84.9	65.4	40.0	0.39***	0.14***
Extended family (some/all %, *n* = 1375)	84.8	65.5	46.6	0.36***	0.17***
Straight friends (some/all %, *n* = 1246)	95.1	86.2	77.2	0.34***	0.19***
Fellow students (some/all %, *n* = 1086)	84.2	71.0	57.9	0.51***	0.32***
Perceived acceptance by					
Mother (completely %, *n* = 911)	66.9	55.5	44.4	0.63**	0.40**
Father (completely %, *n* = 795)^a^	59.1	53.9	.	0.77	.
Extended family (all %, *n* = 914)	77.0	69.3	58.5	0.65*	0.39**
Straight friends (all %, *n* = 1107)	91.5	89.0	86.2	0.73	0.55
Fellow students (all %, *n* = 704)^a^	76.5	70.6	.	0.69	.
Perceived victimization (yes % in preceding year)					
Parents (*n* = 1449)	19.0	20.9	11.8	1.13	0.57
Extended family (*n* = 1449)	11.0	16.9	12.5	1.80**	1.35
Straight friends (*n* = 1449)	21.5	27.6	19.9	1.38*	0.90
Fellow students (*n* = 1213)	30.1	31.9	17.8	1.30	0.67
Suicidality (yes % lifetime)					
Ideation (*n* = 1308)	56.8	52.8	59.6	0.81	1.03
Attempt (*n* = 1308)	11.4	13.4	21.1	1.22	2.20**

^a^In the equally both-sex attracted subgroup, the number of observations was less than 50. Logistic regression was not performed

^b^Not controlled for other variables

^c^Controlled for age, gender, importance, openness, and relationship experience with same-sex partner. Degree of openness was not controlled for in relation to outcome variables on openness and perceived acceptance

*p < 0.05; **p < 0.01; ***p < 0.001

**Table 3:** Results for visibility discomfort and self-esteem

		Mean scores^a^					Linear regression analyses^b^			
		Exclusively same-sex attracted	Mostly same-sex attracted	Equally both-sex attracted			Mostly same-sex attracted versus exclusively same-sex attracted		Equally both-sex attracted versus exclusively same-sex attracted	
Outcome variables	Range	*M* (SD*)*	*M* (SD)	*M* (SD)	*F* value	*df*	*b* (SE)	*p*	*b* (SE)	*p*
Visibility discomfort; *n* = 1449)	0–4 (low to high)	0.99 (0.73)	1.28 (0.83)	1.35 (0.80)	50.42	(6, 1442)	0.24 (0.04)	***	0.28 (0.07)	***
Self-esteem; *n* = 1449)	0–4 (low to high)	2.86 (0.72)	2.68 (0.76)	2.60 (0.79)	21.63	(7, 1440)	−0.05 (0.04)		−0.03 (0.07)	

^a^Not controlled for other variables

^b^Controlled for age, gender, importance, openness, and relationship experience with same-sex partner. Degree of openness was not controlled for in relation to the outcome variable visibility discomfort

*p < 0.05; **p < 0.01; ***p < 0.001

#### Openness

Compared to exclusively same-sex attracted participants, the mostly same-sex attracted and equally both-sex attracted participants were less open about their sexual orientation to their mother, father, extended family, straight friends, and fellow students. There were large differences between sexual attraction subgroups and between contexts. The percentage of exclusively same-sex attracted participants who were open ranged between 84 and 95 % in different contexts, while for equally both-sex attracted participants, this range was between 40 and 77 %. In all contexts, differences between sexual attraction subgroups remained significant in logistic regression analyses (*p* < 0.001 in all contexts).

#### Perceived Acceptance

Differences in perceived acceptance varied between contexts. Mostly same-sex attracted and equally both-sex attracted participants reported less perceived acceptance by their mother and extended family compared to exclusively same-sex attracted participants. In logistic regression analyses, these differences remained significant in the context of the mother (mostly same-sex attracted: odds ratio (OR) = 0.63, *p* = 0.004; equally both-sex attracted: OR = 0.40, *p* = 0.003) and extended family (mostly same-sex attracted: OR = 0.65, *p* = 0.013; equally both-sex attracted: OR = 0.39, *p* = 0.002). In other contexts, the differences were small and were not significant in logistic regression analyses. In the case of the father and fellow students, the size of the equally both-sex attracted subgroup was too small to run analyses, which was related to lower degrees of openness in these contexts.

#### Perceived Experiences of Victimization

Perceived experiences of victimization due to the participants’ sexual attraction by their parents, extended family members, straight friends, or fellow students in the preceding year were most frequently reported by the mostly same-sex attracted subgroup. The equally both-sex attracted participants were least likely to report perceived experiences of victimization. In logistic regression analyses, the mostly same-sex attracted subgroup remained more likely to have experienced perceived victimization by extended family (OR = 1.80, *p* = 0.001) and straight friends (OR = 1.38, *p* = 0.022).

#### Suicidality

Around half the participants in all sexual attraction subgroups reported lifetime suicidal ideation. Differences were negligible and not significant in the logistic regression analysis. Suicide attempts were reported more by equally both-sex attracted participants (21.1 %) than mostly same-sex attracted (13.4 %) or exclusively same-sex attracted participants (11.4 %). This difference remained significant in the logistic regression analysis (OR = 2.20, *p* = 0.006).

#### Visibility Discomfort

Compared to the exclusively same-sex attracted participants, the mostly same-sex attracted and equally both-sex attracted participants reported more discomfort with being open and visible about their sexual orientation. Since visibility discomfort and degree of openness (*r* = −0.65, *p* < 0.001) were highly correlated, the latter variable was not included in the linear regression analysis. In this analysis, differences between sexual attraction subgroups remained significant (mostly same-sex attracted: *b* = 0.24, standard error (SE) = 0.24, *p* < 0.001; equally both-sex attracted: *b* = 0.28, SE = 0.07, *p* ≤ 0.001).

#### Self-Esteem

Equally both-sex attracted participants reported lower scores on self-esteem compared to mostly same-sex attracted or exclusively same-sex attracted participants. In a linear regression analysis, these differences were not significant after controlling for other variables, perhaps because of the fairly strong effects of gender (*b* = 0.22, SE = 0.04, *p* ≤ 0.001) and openness (*b* = 0.18, SE = 0.04, *p* ≤ 0.001).

### Unique Issues for Bisexual-Identified Dutch Young People

Based on qualitative data, participants’ bisexual experiences and views on bisexuality were explored, as well as the space for them to identify as bisexual and to express bisexuality. Several unique issues were identified related to bisexuality or to views on sexual orientation which impacted on the lives of bisexual-identified participants: (a) marginalization of bisexuality through reification of the hetero/homo binary, (b) difficulty expressing bisexuality, particularly in relationships, and (c) a lack of bisexual or bi-inclusive communities.

#### Marginalization through Reification of the Hetero/Homo Binary

Views on bisexuality by non-bisexual-identified SSA participants showed that bisexuality as a sexual orientation was surrounded with controversy, which implicitly reified the hetero/homo binary and consequently marginalized bisexuality as a sexual orientation. A minority considered bisexuality a stable and “real” sexual orientation, similar to homosexuality and heterosexuality. However, more than half the participants considered bisexuality not to be a stable sexual orientation and/or perceived it as a temporary phase. Stereotypes about bisexuality emerged, such as bisexual people not having made up their minds yet or wanting to have it both ways. Several non-bisexual-identified participants did not take bisexuality seriously. According to Paul (man, 18 years): “If someone comes out as ‘I am bisexual’, I always say to them: ‘Well, you’ll find out later … you’re just *homo*’.”

Some participants regarded the label “bisexual” as a temporary state with which they are at ease and comfortable, since it seems less definitive than expressing a homosexual orientation. According to some participants, coming out as bisexual keeps the option of heterosexuality open.

For me, when you say straight away that you are lesbian, that’s a really big step. But being bisexual, I think that’s something in between, between two worlds. When you say it like that, with one foot in each camp, it feels okay. That’s how I felt, anyway. (Xena, woman, 21 years)

However, most people who spoke about bisexuality as a temporary state were also critical because of its indecisive character. As a consequence, some participants expected that bisexual people would ultimately choose between a heterosexual or homosexual orientation. Bisexual-identified participants also spoke about pressure to “know” or “decide” about their sexual orientation, in which case a bisexual status was not considered a valid option.

By then, the entire student association knew. Many of the men gave me reactions like: ’Do you know for sure? Do we still have a chance?’, which was quite annoying because I didn’t know. (Joanne, woman, 21 years)

Joanne felt mostly same-sex attracted, but found it difficult to say she was lesbian because she was not completely sure. The idea of coming out as lesbian and then finding she liked a man was scary for her. This example shows how sexual orientation is predominantly conceptualized as a binary in which the flexibility to switch between sexual orientations is minimal. Other participants also felt that they had “to be certain” about being heterosexual or homosexual^3^ before coming out to others. Several of them mentioned that they had waited to disclose their same-sex sexuality to others because they first wanted to be “sure.” Apparently, they felt a lack of space to share or discuss their early sexual orientation awareness, or else they had doubts or felt that bisexuality was not an option. Young people who identified as bisexual also expressed how sexual orientation was conceptualized as a binary, consisting of a heterosexual and homosexual option. Three out of seven (Linda, Tamara, Ilan) perceived a homosexual orientation to be the “default” for same-sex sexuality.

#### Space Limited to Temporary Bisexuality and Women’s Sexual Behavior

In daily life, bisexual-identified young people have to find acceptable ways to frame and express their bisexuality. Some of the bisexual-identified participants embraced their bisexual orientation, but none of them were open to all their friends and family. While there was not much space for a bisexual identity as a “serious” sexual orientation, there were two exceptions in which bisexuality was allowed or more likely to be tolerated: (a) temporary bisexuality and (b) sexual behavior between women framed as bisexual or bi-curious.

Temporary bisexuality refers to bisexuality as a temporary phase en route towards a homosexual or heterosexual orientation. The notion of temporary bisexuality provided young people with the space to experiment with their sexual orientation. However, this option did not create space for a bisexual identity and a bisexual orientation was not fully acknowledged. Young people could feel pressured to ultimately “choose” between a homosexual or heterosexual orientation.

There seems to be space among young people, and especially in specific settings such as college life, for sexual behavior between women that is framed as bisexual or bi-curious (i.e., people who have a curiosity or interest in sexual behavior or relationships with more than one gender, but who do not identify as bisexual). Among the participants, examples were mentioned where sexual behavior between women was permitted or even encouraged, as long as it was exciting for men or at least did not make them feel threatened or excluded. Claire’s account showed how she perceived having more space for a bisexual than a lesbian identity in the context of her student community. She felt mostly same-sex attracted, yet labeled herself as bisexual to other people:

Lesbian … it sounds as if you don’t want to have to do anything with men. It just sounds so final. I have just fought so long to not label myself like that, but I still have difficulties with that word. […] It’s very strange but I think, yes, you are it [lesbian], but you just don’t call yourself that. That’s just the way it is. […]. Bi, well that’s exciting and fun, while lesbian is immediately: “Whoa!” They then speak to you differently, and that’s a pity. (Claire, woman, 21 years)

Thompson (2006) noted that a lesbian position does not leave a role for men and excludes young women from both male and female peers who endorse heterosexuality. Like Claire, several other young women mentioned that coming out as lesbian distanced them more from their heterosexual peers than coming out as bisexual. She knew several heterosexual young women who had had sexual encounters with other women without questioning their heterosexual orientation. Women’s sexual behavior framed as bisexual or bi-curious was reconcilable with heterosexuality since it did not leave men out of the picture as potential sexual or relational partners. These bisexual or bi-curious sexual expressions provided behavioral space for young women to engage in sexual exploration in adolescence; however, they did not consider bisexuality to be a stable or serious long-term position or identity. Thus, the hetero/homo binary was left intact.

There was less space for a bisexual identity and bisexual expressions among men. Positive experiences and attitudes towards sexual behavior between men framed as bisexual or bi-curious were not brought up by any of the participants. In line with other Dutch studies (Felten et al. 2010; Van Lisdonk and Kooiman 2012), young men seemed to feel more pressured than young women to conform to the hetero/homo binary. With the exception of Tom, young men felt uncomfortable acknowledging that they felt (or had at some time felt) attracted to both men and women. They seemed to prefer an exclusively homosexual or heterosexual orientation. Several young men reported that they had experienced a “bisexual phase” prior to their homosexual orientation. Yet, a bisexual identification was undesirable for them. Ilan perceived himself as bisexual, but wished he was unambiguously homosexual or heterosexual. Erik and Stephen felt ambiguous about interpreting their past crushes on young women, since they preferred a homosexual orientation. They did not label themselves as bisexual to other people.

#### Relationship-Building and Expressing a Bisexual Orientation

Space to come out as bisexual was particularly limited when people were in a long-term relationship with one partner. The three participants, Ilan, Linda, and Nicola, who identified as bisexual and who were in long-term relationships with one partner, generally chose to pass for heterosexual or homosexual, according to the gender of their partner. Ilan (man, 25 years) was too uncomfortable to disclose his curiosity and slight desire for women to his friends and to his partner, who was a man. They had the impression that his sexual orientation had changed from bisexual to gay since he had a long-term relationship with this partner. He described his situation as “a kind of reversed being in the closet”: “The word in all situations is actually that I have a boyfriend. […] I only implied that I have a boyfriend and then people assume that you are ‘homo.’ And as for bi, that doesn’t exist.”

While Ilan passed for gay, Linda and Nicola, who were both in long-term relationships with men, usually passed for heterosexual. Both women were comfortable with their own bisexuality, yet were selective in their openness to others. According to 24-year-old Linda, it was important for her to be open about her bisexuality since openness and honesty would increase acceptance. However, her family and friends did not take her bisexuality for granted the way she did. Her mother considered her bisexuality to be a trial, or a past phase, since Linda now had a boyfriend. Lesbian friends asked her: “What’s it like with your boyfriend, and how is it to go back to a man?” Linda was not open to her boyfriend’s circle of friends and family, who were all heterosexual, because her boyfriend preferred her to be cautious: “I want my friends to have a good impression of you first, before they get prejudiced.” And he also said, “I would find it strange if you talked about women all the time.”

Nicola, 21 years old, was also selective in her openness about her bisexuality in heterosexual contexts. In her neighborhood and daily life, people thought she lived an ordinary life and was in a long-term heterosexual relationship. However, that was not her whole story: “We live a kind of a double life. At home, we live as a little family. But in addition, we have our erotic parties with swinging and friendships with an erotic element.” Nicola’s mother and sister were the only non-swinging straight people who knew about her bisexuality and polyamorous lifestyle. She explains her reticence to be open:

Look, if I’d come home with a woman or girlfriend, you know, then I’d have said to my family: ‘Listen, it’s a not a he but a she.’ But that’s not how it was. So then it’s really strange to say, like, ‘Well, I have something to tell you: I live with Curt and that’s all going fine, but I’m bi.’ That feels strange.

Each of these three participants had found a way to express their bisexuality within their long-term relationship. Linda and Nicola had an agreement with their partners that they were allowed to occasionally hook up with a woman under specific conditions. Ilan mentioned with some embarrassment that he had secretly visited a female sex worker. Relationship-building for young people who were attracted to women and men to some extent posed challenges. In this small sample, there were no bisexual-identified participants who firmly preferred serial monogamy (i.e., preferring relationship or sex partners sequentially, Rust 2002) or preferred casual sex and no relationships.

#### Lack of Bisexual or Bi-inclusive Communities

The hetero/homo binary is not only the obvious norm among heterosexual people, but also among many lesbian women and gay men (Ault 1996; Barker et al. 2012a; Rust 2002). Bisexual-identified participants did not perceive their lesbian and gay friends to be more open-minded or accepting of bisexuality than their heterosexual friends.

Bisexual-identified young women, in particular, did not feel completely at home in LGB public venues, which they perceived more as lesbian and gay oriented. These young women, as well as feminine-looking young women, reported having been confronted with responses from lesbian women that they did not look “lesbian enough.” This made them feel marginalized and excluded. The bisexual-identified young men did not have similar complaints about not feeling at home in LGB public venues.

The young men did not address bisexual organizations or public venues. For young women, there were a few online and offline venues. According to Nicola, who has visited some venues, these bisexual communities merely promoted one specific kind of bisexuality:

[talking about bisexual communities] Well, I find it a real teen scene. It’s very disappointing. The funny thing is, at [name website and parties for bisexual young women] … I have been there and then you find it’s all chicks of 16 or 17 with their boyfriends. I find that odd: All bi, but all with a boyfriend. Something isn’t right, know what I mean? And then you see a man who asks someone: ‘Fancy kissing my woman?’ Well, that’s kind of the level of things there.

These communities could be interpreted as promoting *performative bisexuality*, centered around a *male gaze* (Thompson 2006).

Nicola had difficulty in feeling connected and at home in any community: The bisexual communities she knew were predominantly organized around a narrowly defined form of bisexuality, while she found lesbian communities not open towards bisexuality. She liked the notion of polyamory and together with her partner they became part of swinging communities. However, she realized that swinging communities only encouraged other-sex sexual behavior, and same-sex behavior between women, which convinced her that bisexuality as a real sexual orientation was not fully accepted.

## Discussion

This study shows that it is relevant to distinguish between subgroups within the larger population of SSA individuals. Generally, Dutch equally both-sex attracted young people reported more negative experiences than their exclusively same-sex attracted counterparts. They were less open to people in their social network, reported less perceived acceptance by their mother and extended family, and scored higher on visibility discomfort and suicide attempts. Equally both-sex attracted young people did not differ in degree of self-esteem, suicidal ideation, or perceived experiences of victimization. It was useful to control for other variables since seemingly large differences between the exclusively same-sex attracted and equally both-sex attracted subgroups on self-esteem and perceived experiences of victimization in specific contexts disappeared, which implies these differences were accounted for by other factors. It was relevant to separate the mostly same-sex attracted participants from the other two subgroups because the outcomes for this subgroup differed, often lying between those for the exclusively same-sex attracted and equally both-sex attracted subgroups.

This study demonstrates that a bisexual orientation is marginalized rather than irrelevant in people’s lives. While in the survey, the equally both-sex attracted subgroup reported lower scores on openness and the importance of their sexual attraction in their lives, this study provides indications that their invisibility is not so much a consequence of a lower relevance or desire to be open, but is related to feeling that there is a lack of space in society to be open. The lower degree of openness and higher degree of visibility discomfort remained significant after controlling for the importance of sexual attraction, having experience with same-sex partners and being younger. These participants also generally felt less accepted compared to exclusively same-sex attracted participants.

In the interviews, all bisexual-identified participants mentioned difficulties in disclosing their sexual orientation to others, also to same-sex attracted friends and partners. All bisexual-identified participants used strategies to help them feel more included and accepted in heteronormative or lesbian/gay-normative contexts, such as passing for lesbian/gay, and *covering* (i.e., making a great effort to adjust and assimilate, even though others knew about their bisexuality, Goffman, 1963). The space for a bisexual identity and for bisexual expressions was limited to those forms that did not challenge heteronormativity and the hetero/homo binary. There was space for temporary bisexuality and same-sex sexual behavior between women framed as bisexual or bi-curious. However, a bisexual identity or bisexuality as a valid long-term sexual orientation was met with rejection or suspicion.

Several specific issues for bisexual people were identified as follows: The marginalization of bisexuality, difficulties in being open and expressing bisexuality, particularly in relationships, and a lack of bisexual or bi-inclusive communities. They were well aware of bisexuality being associated with non-monogamy, not being able to be a good partner and not yet having made a choice. They attempted to strike a balance between being true to themselves and risking causing harm to social and intimate partner relationships by openly expressing their bisexuality. The lack of bisexual or truly bi-inclusive public venues imparted a sense of not belonging to a community for several bisexual-identified participants.

The specific issues which were addressed were related to the hetero/homo binary and to mononormativity. The marginalization, and therefore invisibility, of bisexuality in Dutch society is related to the way sexual orientation is conceptualized: As a hetero/homo binary in which sexual orientation is perceived in an essentialist way, implying that heterosexuality or homosexuality are the primary options (Maliepaard 2015a, b; McLean 2008; Richardson and Monro 2012; Rust 2002). In such a social climate, bisexuality is neither acknowledged nor visible (Maliepaard 2015a, b; Rust 2002). Rather, it is silenced and denied (Maliepaard 2015a; McLean 2008; Rust 2002). The American scholar Robinson came to a similar conclusion and noticed that in interviews with Dutch LGBT adults about Dutch LGBT communities, bisexuality was not discussed at all (Robinson 2012). He stated that the “erasure of a bisexual identity” is not surprising in a society in which sexuality is constantly reified within a hetero/homo binary discourse (Robinson 2012, p. 335).

At the same time, their stories also show that monosexuality (i.e., sexual attraction focused on only one gender: heterosexuality and homosexuality) is the norm in society. In a mononormative society, bisexuality is framed in opposition to heterosexuality/homosexuality, thus creating distance between these sexual orientations and fostering stigma and stereotyping (Ault 1996). Bisexual-identified young people often attempted to present themselves as monosexual in order to foster acceptance.

In the literature, there are two seemingly contrasting perspectives on the space for bisexual identity and bisexual expressions among young people. There is an assumption about young people being relatively rigid about nonconformity in relation to gender and sexuality (Lobel et al. 2004), which may also apply to the space for a bisexual identity and bisexual expressions in the societal context of a dominant hetero/homo binary. In contrast, other scholars noted evidence and space for sexual fluidity, particularly among young women (Dempsey et al. 2001; Diamond 2008; Savin-Williams 2005; Thompson, 2006), which may also suggest space for a bisexual identity and bisexual expressions. We found evidence for both assumptions. Generally, the young SSA people in this study appeared to be rather rigid in their conceptualization of sexual orientation as hetero/homo binary. Without exception, they perceived gender to be binary and none of them questioned or played with this notion of gender.

However, the two contexts in which bisexual expressions were permitted or tolerated both demonstrated sexual fluidity. Bisexuality as a temporal phase in young people’s sexual development shows that sexual orientation is not always fixed or clear, and confirms the view that sexual orientation is a continuum and may change over time (Diamond 2008). Same-sex sexual behavior between women framed as bisexual or bi-curious provides space for young women’s desire to fully explore their sexuality beyond their sexual self-identification or labeling.

These spaces for a bisexual identity and bisexual expressions were regulated within the constraints of the hetero/homo binary, heteronormativity, and monosexuality: Both young men and women felt pressured to be sure about their sexual orientation, which implied a homosexual or heterosexual identity. A bisexual phase, questioning, changing, and same-sex behavior between women framed as bisexual or bi-curious was allowed as they were young and at the beginning of their sexual development, but they were expected ultimately to choose between a heterosexual or homosexual orientation. We also note that the space for a bisexual identity and bisexual expressions was more limited for young men and that young men were generally more uncomfortable about their bisexual experiences.

### Implications for Bi-inclusive Policies

The empirical findings provide four pointers for more bi-inclusive policies: (a) awareness-raising concerning marginalization, visibility and bi-specific issues; (b) community and capacity building; (c) comprehensive policy based on gender and sexuality diversity; and (d) comprehensive sexuality education. An important first step is to promote awareness of marginalization processes in relation to bisexuality, promote the visibility of bisexuality, and draw attention to specific issues which are typically relevant to bisexual (young) people, such as the existence of biphobia and recognition of diverse desires in terms of relationships and challenges for bisexual people in feeling a part of a community. A proportion of Dutch young SSA people clearly expressed biphobia and they did not always perceive bisexuality to be a serious and valid long-term sexual identity. In a society in which bisexuality was valued just as much as homosexuality, including among SSA people, Dutch young people would probably not be so cautious or uncomfortable in disclosing a bisexual orientation compared to a homosexual orientation. Ministries should screen their own language use in policies on biphobia and implicit marginalization of bisexuality, and should explicitly draw attention to the recognition of bisexuality and bisexual people in lesbian and gay communities and wider society in order to reduce biphobia (Felten and Maliepaard 2015). In addition, interventions, activities, and research funded by the government could be screened for bi-inclusiveness that goes beyond lip service and to avoid mononormativity. For example, *gay-straight alliances* are government-funded interventions which are developed in several social domains. Yet, the language used clearly marginalizes bisexuality.

Second, promoting community and capacity building of bisexual communities and bi-inclusiveness of LGBT communities is important. Young bisexual-identified people did not feel welcomed in LG(B) communities, nor did they find their way to bisexual communities, while this study showed that both-sex attracted and bisexual-identified young people considered their sexual orientation to be important. The bisexual-identified participants felt marginalized and forced to adjust to living in heteronormative and homonormative communities. The only reference to bisexual communities was a community for bisexual young women which was implicitly heteronormative, with a focus on the male gaze. The young bisexual-identified people in our study did not engage with organizations explicitly focused on bisexual people, such as the largest bisexual national network and interest group for bisexuality, LNBi, nor Orpheus (a support organization for homosexual and bisexual people in man/woman relationships), or other organizations and initiatives focused on bisexuality, sexual fluidity, polyamory, and queerness. The well-established and largest Dutch LGBT organization, COC Netherlands, has recently started to organize initiatives to foster bi-inclusiveness; while this is promising, COC Netherlands is still largely associated with homosexuality.

Although participants in this study did not address the need for community and capacity building to increase societal visibility and political representation of bisexual people, this strategy has been proven to be successful for other marginalized subgroups. In the Netherlands, government funding of the transgender movement has increased the capacity building of this community and the visibility of transgender people in society, which has consequently increased the representation of transgender people in policy. Even though bisexual-identified young people may not be interested to become active in a bisexual community, they might still benefit from strong, diverse, visible, and bisexual communities which promote societal visibility of bisexuality and which offer information, support, and identification.

Dutch authorities can contribute to reducing marginalization and increasing the visibility and perception of bi-specific issues by fostering the representation of bisexual and bi-inclusive communities and experts at the same time as they consult gay- and lesbian-focused communities and experts (Felten and Maliepaard 2015). In doing so, they do need to be conscious of which bisexual people they consult and are represented—including young bisexual people—and which people remain marginalized (e.g., queer or polyamorous people, bisexual people in monogamous other-sex relationships, bisexual people with a migrant or non-middle class background or living in rural areas).

Third, bi-inclusive policies will not be successful if the underlying goal remains focused on equality and emphasizing sameness. This study showed that specific issues of bisexual-identified young people were related to the unchallenged hetero/homo binary and mononormativity. In a sexual minority approach based on sameness and normalization, issues which specifically matter to bisexual people are difficult to bring to light and to place on the political agenda, since the hetero/homo binary and mononormativity are perpetuated and not critically questioned. Furthermore, in line with other scholars, we conclude that heteronormativity and related views on sexuality and gender are not challenged in Dutch society and politics (Maliepaard 2015a; Mepschen et al. 2010; Robinson 2012). As a consequence, people who do not fit into a heteronormative framework are likely to be less represented and remain structurally marginalized in such LGBT policies (Maliepaard 2015a).

Hence, we note that comprehensive bi-inclusive policies require a policy perspective focused on gender and sexual diversity. A diversity perspective that recognizes differences and diversified experiences and identities because of intersections promotes visibility of differences and gives voice to being different (Ghorashi and Ponzoni 2014; Richardson and Monro 2012). This leaves space for a diversity of experiences and expressions of sex, sexuality, and gender as non-binary, fluid, or genderless, which may also impact on views and expressions in terms of relationships and “the family.”

In a society which values diversity, processes of normalization in terms of power indifferences and bias can be critically explored and questioned (Ghorashi and Ponzoni 2014). Policies could focus more on revealing and reducing marginalization of specific sexual, gender, and relational expressions and identities, based on restrictive normativity and stereotypes grounded in the hetero/homo binary, monosexuality, and the conceptualization of sex and gender as binary. Instead of current fragmented policies targeting inequality for women (predominantly focused on the labor market and gendered labor/care division) and LGBT people (predominantly focused on promoting acceptance and reducing stigmatization), an integrated diversity perspective might open up the political arena for raising new questions and issues. New issues which might be particularly beneficial for bisexual people are the social and legal recognition of plurality in relationships, including polyamorous partnerships; acknowledgement of non-binaries; and fluidity in sexual orientation, gender identity, and expression.

A diversity perspective on sexuality and gender could be particularly beneficial for young people who are exploring their sexuality and may experience doubts and changing desires. In a society which recognizes sexual and gender diversity and fluidity, pressure to know or choose a sexual orientation would decrease and it would be easier for (young) people to speak about their desires, doubts, and worries with others, which might enhance the likelihood of developing a strong (sexual) self. Moreover, they may feel more comfortable in perceiving their sexual orientation and gender as beyond binaries.

Finally, policies based on diversity may encourage comprehensive sexuality and gender education based on the above-mentioned notions. Clearly, these SSA young people expressed binary conceptualizations of sexual orientation and gender. In 2012, the Dutch government introduced attainment targets for Dutch schools in relation to sexuality and sexual diversity. While schools are free to teach their own views on sexual diversity, in funding school education programs, the government could formulate a basic set of topics and a minimum of research-based information, including information on bisexuality and gender diversity.

### Limitations and Research Suggestions

A limitation of this study was the use of a convenience sample, which may have drawbacks regarding the sample composition. In the Netherlands, it is almost impossible to draw a representative sample within this age range which is large enough to distinguish between sexual attraction subgroups among SSA people. In anticipation of possible drawbacks, we used offline and online recruitment channels, aiming at young people in general as well as LGB young people and bisexual young people in particular. Nevertheless, recruitment through self-selection and self-report may have attracted participants who are relatively open about their sexual orientation, who affiliate with LGB communities, and who are less likely to be bisexual oriented (Saewyc et al. 2009). In future research, a better option would be to use large general research panels in which possible sample selectivity due to LGB-related recruitment is minimal (Herek 2009; Kuyper 2015a; McDermott 2010).

Moreover, more in-depth research among bisexual people, with a particular focus on those who do not identify with or participate in LGB communities, would be interesting. Our qualitative sample was rather small and did not include the full range of the diverse stories of bisexual people. The interviewees in our study viewed gender as a binary. There were no or few interviewees who preferred serial monogamy, casual sex, no relationships, and none identified as queer, gender fluid, or transgender. These stories might provide new insights on limited space in sexuality and gender diversity, and consequently additional policy recommendations.

Unfortunately, we were unable to distinguish between equally both-sex attracted young men and women in the survey. Since the quantitative sample consisted of a relatively small number of equally both-sex attracted men, the results may not have captured gender-specific issues for this group.

To reduce marginalization of bisexuality and foster a better understanding of the experiences of bisexual people, we encourage researchers to always be explicit about measuring sexual orientation and gender. They should also not assume self-evident links between sexual attraction and identification. In the case of sexual attraction, researchers should distinguish between different attraction subgroups, if possible. Furthermore, assessing non-binary choices of gender identity in large-scale survey studies requires attention.

Finally, the quantitative data presented here were a subset of a larger survey, which addressed a broad range of topics. Therefore, extensive measurement of topics such as internalized homo/biphobia or composition, quality, and quantity of social networks was not possible. More in-depth studies on attitudes towards bisexuality and bisexual expression, and the impact of biphobia could provide a better insight into the origins and processes of marginalization. A gender perspective is useful here. Herek (2000) noted that attitudes are likely to be different towards lesbian women, gay men, bisexual women, and bisexual men, since social interaction may impact on individuals’ own sexual and gender identity. This suggests that a gender and sexual diversity perspective on stigma and normativity might provide better insights than a sexual minority perspective.

## Conclusions

Our aim in this article was to place on the academic and policy research agenda the idea that it is important to distinguish consistently between homosexuality and bisexuality, to be aware of diversity in experiences and to develop bi-inclusive policies. While there is a growing academic and political awareness that attention for bisexuality is needed, there are still few good practices regarding what bi-inclusive policies should look like. Based on our study, we argue that policies should focus on awareness of marginalization processes and invisibility of bisexuality, specific problems bisexual-identified people encounter, insight into and attention to biphobia, community and capacity building, and comprehensive sexuality and gender education. Many of the reported difficulties for both-sex attracted and bisexual-identified young people were related to a conceptualization of sexual orientation as a hetero/homo binary and the norm of monosexuality. We would suggest that a policy model which focuses on sameness and normalization is not likely to challenge the hetero/homo binary and mononormativity, and therefore cannot be truly bi-inclusive. A model based on a more comprehensive perspective on gender and sexual diversity could offer more space for bisexual identities and expressions. This would not only be beneficial to bisexual people, but also to all those, and especially young people, who are exploring their sexuality and developing their sexual self.

## Footnotes

1 The only exception was the English version of the 2007/2008 policy document, titled “LGBT policy plan,” which resonated with upcoming international terminology trends.

2 Explicit visibility of or attention for transgender people is also limited. However, there is an increasingly visible transgender movement and a growing awareness among LGBT activists and organizations that transgender people have unique problems and issues which require attention.

3 In the Netherlands, the label ‘homosexual’ is not considered derogatory and can be used in a neutral way.
